# Factor analysis of key parameters for effective design delivery of urban transport infrastructure in Ethiopia

**DOI:** 10.1016/j.heliyon.2024.e34681

**Published:** 2024-07-15

**Authors:** Leule M. Hailemariam, Denamo A. Nuramo

**Affiliations:** Ethiopian Institute of Architecture, Building Construction and City Development (EIABC), Addis Ababa University (AAU), Addis Ababa, Ethiopia

**Keywords:** Effective design approach, Factor analysis, Design process, Transport infrastructure, Urban infrastructure, Ethiopia

## Abstract

Urban infrastructure can be depicted as critical facilities for providing urban public services and supporting urban socioeconomic activities. Economic growth, especially the provision of sustainable mobility in urban areas, is found to be reliant on the availability of effective transport infrastructure. Urban transport assets, mainly road and railway infrastructure, have notable influence on the safety and convenient connectivity of economies, communities, and ecosystems. While the vitality of urban transport infrastructure provision is well recognized, its practice is marred by several challenges. Addressing those challenges may require interventions throughout the life cycle of the facilities, including planning, design, construction, commissioning, operation, and demolition phases. This study, however, focuses on formulating key guiding principles and methods for effective design of transport infrastructure in urban areas of Ethiopia. The provision of transport infrastructure requires an effective design methodology throughout the lifecycle of the built element. The study involved a thorough analysis of the literature review to identify the current delivery approaches, and a questionnaire survey with a sample size of 204 (N = 204) was then utilized to determine, based on the opinions of experts, the most efficient methods of delivering transportation infrastructure. A descriptive and factor analysis were utilized to forward the demographic data of the research findings and to identify the key components of transport infrastructure delivery through design, respectively. The study found that the principal components that directly impact the design and delivery process of urban transport are design context, design necessities, institutional competency, and professional competency. It can be concluded that consideration of the principal components at the time of design assignment would facilitate the design products' effectiveness.

## 1 Introduction

Sustainable transportation and economic growth are fundamentally dependent on effective infrastructure [1]. Urban infrastructures are critical facilities for providing urban public services and supporting urban socioeconomic activities, and they include a wide range of functional infrastructures such as water, gas, electricity, telecommunications, and transportation infrastructure [2,3]. Transport infrastructure is essential for public connectedness, which creates an input for society's development. Adequate and high-quality transportation networks improve the efficiency with which social infrastructure such as healthcare, education, public amenities, and transportation are linked. Social infrastructure can provide significant benefits including faster economic growth, increased productivity, poverty, alleviation, and environmental sustainability. The social sector, which includes education, health and nutrition care, housing, and the water supply, is critical for social and economic development. On the other hand, as stated by Ref. [4] as a basic model, when city populations are low or institutions are strong, private provision of infrastructure to prevent negative externalities is less expensive, whereas public provision can be less expensive in larger cities. Western cities grew up in largely closed economies, where local agricultural production and transit technology were required to feed urbanites. Glaeser (2014) also attempted to investigate the governance of large cities with weak institutions. A frontier of institutional possibilities evolved, reflecting the combination of city population and institutional excellence. Larger cities are more likely to require government involvement to mitigate externalities, but urban anonymity makes enforcing good results more difficult, especially when weak institutions frequently make bribes [4,5].

The transportation infrastructure requires a comprehensive design, unified layout, and rational adjustment of the transportation structure [6]. First, infrastructure development should optimize transportation and increase the efficiency of transportation networks. Second, the government responsible for devising strategies and policies should manage the fragmentation of urbanization. A coordinated urbanization strategy requires equalization of public resources in the region, as well as effective improvement of urbanization quality, to enable the achievement of transportation efficiency [6]. Developing ecologically friendly transportation systems is a key challenge for countries worldwide. Transport infrastructure delivery systems are most likely to be integrated project delivery trends, implying delivery through collaboration and integration [7]. The provision of urban transport infrastructure is not an island without any linkage to neighboring rural areas; thus, effective delivery of this infrastructure is vital for urban and urban-rural linkages. This transport infrastructure facilitates the mobility and migration of goods, finance, and humans [8]. Transport infrastructure assets are a strong cornerstone of an economy's social and economic development [9].

Roads and rails are the two main transport infrastructures in metropolitan areas, and these vital infrastructures have a significant influence on the safety and convenient connectivity of economies, communities, and ecosystems. Roads are typically provided by the public sector, principally by the government, although roads can also be supported by private sector investment loans. Road development and improvement approaches may be based on technical planning models that attempt to assign costs and benefits to expenditures [10]. It can also help assess the disadvantages of poor accessibility using specific criteria. Railway service delivery varies by country. However, this is not different from the highway delivery systems. However, each infrastructure system has its own delivery mode. The need for effective transportation systems is enormous [11]. Due to rapid urbanization and population growth, the current road and railway infrastructure in Ethiopia, both in terms of network length and breadth, is in great demand [12]. Transportation technology goals, as well as economic and cultural considerations on how to make better use of urban space, have all had an impact on all cities [13].

The importance of designing service delivery systems has also been identified because there is a lack of theoretical development in the field [14]. Design delivery for physical infrastructure revolves around design activity or practice to obtain a useable structural element. Moreover, the effective design delivery mechanism is an effective parameter to measure how transport infrastructure, such as roads and railways, function to deliver the intended design purpose.

According to Rodrigue (2020), urban density is the deciding element that influences the availability of social possibilities such as access to services and commodities, employment, and social interactions, and economic opportunities at constant accessibility/distance which are access to customers and suppliers. At constant accessibility, a high-density urban region will have a higher degree of accessibility than a low-density area and vice versa [13]. Accessibility is a key measure of how successfully public transportation and the built environment are linked. The concept of accessibility, defined here as the ease or possibility of reaching the desired destination from a specific location in space by a certain mode of transportation, can provide a solid foundation for integrating transportation and land-use planning [15]. Considering travel objective, trip time, and travel cost as fundamental pillars of the concept, Bertolini et al. (2005) defined accessibility as: “the amount and diversity of places of activity that can be reached within a given travel time and/or cost”. Abreha (2007) emphasizes that accessibility is a far wider term than mobility, which can completely explain movement patterns, and defines it as “mobility for opportunities” [16]. Cities are areas with high levels of economic activity, accumulation and concentration. They are complex spatial structures supported by infrastructure, such as transportation networks. The greater the complexity and potential for disruption in a city, the greater its complexity, particularly when it is not successfully controlled. The effectiveness of a city's transportation infrastructure in moving workers, customers, and freight between various origins and destinations is critical to productivity [13].

The major issue that needs to be addressed during the delivery of transportation infrastructure in the urban environment at the time of temporary or permanent migration is a population-resource imbalance, which is prevalent in both rural-urban and urban–urban streams [17]. The issue of population–resource imbalance cannot be the only issue or source of problems. There is also the issue of sustainable infrastructure project delivery, in which urbanization or migration should not dictate the delivery of adequate, equitable, and efficient project delivery through planning, design, and construction processes or stages. However, it is a broader problem of how and why an efficient transportation infrastructure delivery system is required. The case of Ethiopia requires more attention and mechanisms because of the city's multifaceted concerns, such as political instability in the city and its surroundings, limited raw material resources, concerns about land use issues, economic issues, and timely and on-budget infrastructure delivery challenges, as well as limited privately financed infrastructure.

Therefore, the delivery of transport infrastructure through design work in making it effective to create sound accessibility and convenient mobility would be the subject of research. This study identifies key mechanisms or approaches to the effectiveness of transport infrastructure in the urban regions of Ethiopia.

To create effective transportation infrastructure that is crucial for the connectivity and mobility of people and goods, it is necessary to identify critical components. An expert survey of those specializing in the design of urban transportation infrastructure would choose the most pertinent and efficient implementation mechanism. What are the most important design delivery factors that should be carefully considered before design work begins in Ethiopia's urban transportation infrastructure? This study establishes the theoretical underpinnings of the delivery system for the urban transportation infrastructure as a whole and its useful characteristics for developing a design delivery system. The factors are reduced using principal component analysis, and the important components of effective design delivery are then identified.

## 2 Method

A survey was used to gather data for the identification of effective delivery tools, and a Likert scale [18]structured questionnaire was employed to collect data, because these types of questions target the level/degree of parameters towards the stated issues. The scales used ranged from 1 to 5 (1 = not important, 2 = low importance, 3 = neutral, 4 = important, and 5 = very important). The questions in the form of factors were collected from the literature in a manner that complied with the main objective inquiry dealing with the effectiveness and design delivery process situation of transport infrastructure.

The characteristics of the respondents' perceptions towards assessing and identifying effective delivery mechanisms are organized based on professionals' involvement in the design of urban transport infrastructure. The selected respondents had design experience with the provision of urban infrastructure. These respondents would have been from different areas, such as financiers, politicians, planners, architects, and engineers, but since the study focused on physical infrastructure design delivery, it was purposefully chosen to include only planners, architects, and engineers.

The sample size (n = 204) was computed using a numerical computation of a 50 % representation of the assumed population proportion (N = 2532), with a margin of error of 10 % and a confidence level of 95 %. The estimated number of registered professionals and practicing urban planners, architects, urban designers, bridge engineers, and structural engineers in the Ministry of Ethiopian Urban Development and Construction works (N = 2532). The population count was obtained in 2021 from the database of the Construction Works Authority. The most important parameters were rated according to the research objective.

The analysis was performed using Statistical Package for the Social Sciences version 23 to quantitatively analyze the data. IBM SPSS (Statistical Package for the Social Sciences) was employed for data analysis because of its ability to determine quantitative data of expert opinions [19,20]. A descriptive and factorial analysis [21] was utilized to identify and reduce the most relevant and vivid main parameters for delivering the transportation infrastructure. Descriptive statistics through percentages and frequency method were used to forward the demographic data of the research findings. Factorial analysis was conducted to identify the key components of transport infrastructure delivery through design. Factorial analysis is used to reduce large parameters or factors to identify effective or key attributes [22]. Principal component analysis was used to reduce the impactful factors that result from multilevel loading [23,24].

## 3 Results

### 3.1 Demography

The general respondents of the questionnaire survey were mostly experts with professional design experience in urban infrastructure. Planners, architects, and engineers responded. The sample characteristics of respondents to the survey questionnaire are depicted in the following demography. The respondents were asked to state their areas of their design experience, and years of design experience in general.

**Fig. 1:**
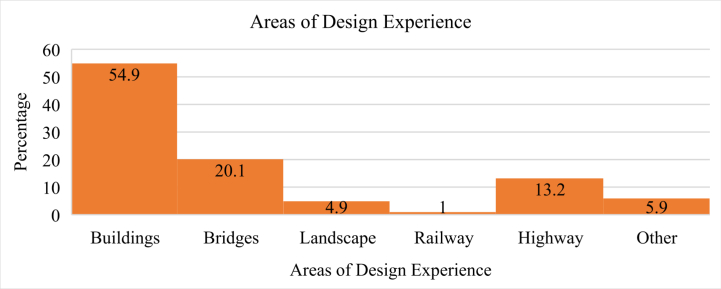
Areas of design experience.

Fig. 1 shows the distribution of the areas of design experience based on major participation in professional practice. 54.9 % designers design buildings, 20.1 % designed bridges, and 13.2 % practiced highway infrastructure design. Landscape designers and other infrastructure planners accounted for 4.9 % and 5.9 % of the total sample size, respectively.

**Fig. 2:**
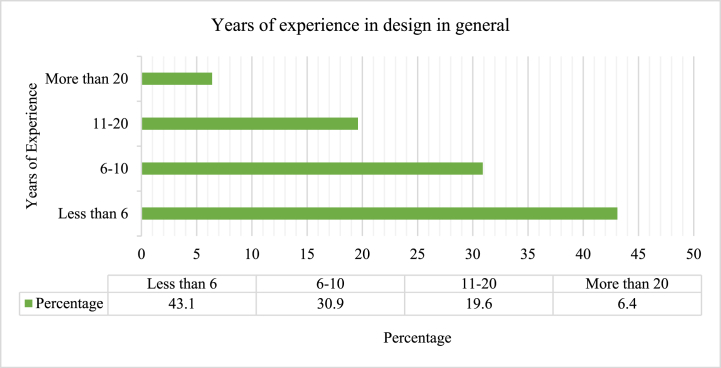
Years of experience in design in general.

Fig. 2 illustrates the general years of respondents’ design experience. A greater percentage (43.1 %) falls in the category of fewer than six years of experience, and this shows that professionals are not engaged in infrastructure design because we need more experienced designers of infrastructure, but it also shows that professionals are interested in the design of infrastructure. On the other hand, we lacked experienced designers with more than 20 years of experience (6.4 %) in taking the representation of these samples. The remaining categories, which were in the practicing professional category, had 30.9 % for 6–10 years and 19.6 % for 11–20 years of experience.

### 3.2 Factorial analysis

#### 3.2.1 Data statistics

The corresponding effective delivery attributes of urban transport infrastructure were identified using a survey distributed to 204 experts and practitioners. The results are then presented in the following section.

The first activity in the analysis of the survey data was to determine its internal consistency of the survey data. Internal consistency can be measured using the reliability of the data collected through a questionnaire. Data regarding the identification of attributes that need to be considered in the provision of urban transport infrastructure.

Therefore, the measure of data reliability is performed using the Cronbach's alpha test [25]. for this case(α = 0.884) shows that the questionnaires have high internal consistency and a high degree of reliability in identifying effective delivery systems of the transport infrastructure.

For a given questionnaire to be considered to have enough internal consistency, the Cronbach's alpha value must be at least 0.70 [26]. For a specific questionnaire, a low Cronbach's alpha value (below 0.7) indicates weak internal consistency and thus poor inter-relatedness across questions. It is discovered that the Cronbach's alpha value depends on the length of the questionnaire (i.e., the number of questions in the questionnaire) and that it rises as the length and subsequently the number of items do [27].

The research questions were organized in a Likert-scale system of measurement of scale of 1–5. The level of importance taken was 1 = not important, 2 = low importance, 3 = neutral, 4 = important & 5 = very important. The parameters in terms of their importance in the identification of effective mechanisms about delivering urban transport infrastructure (in planning and design) were collected from the literatures and then were sent to be rated by the respondents. Effective Delivery Mechanisms (EDM) of urban transport infrastructure are meant to be effective parameters to measure how urban transport infrastructures such as roads and railways function to deliver the intended purpose.

**Fig. 3:**
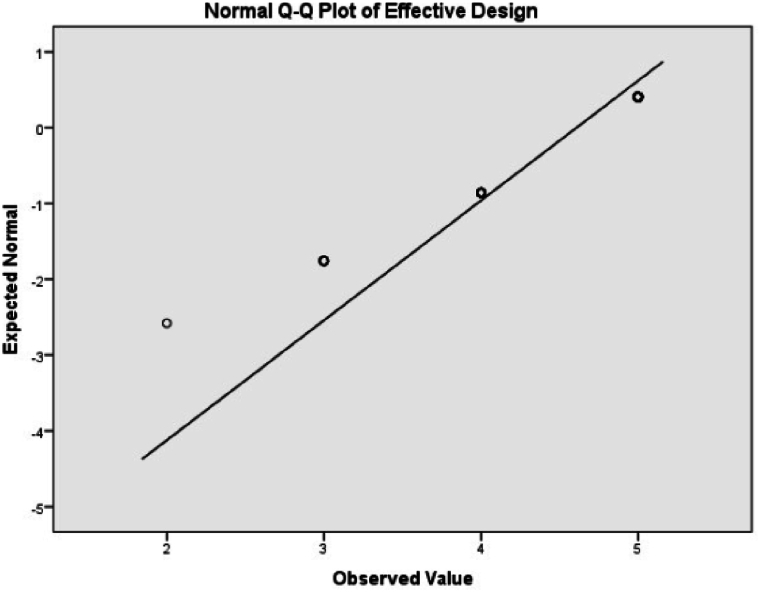
Q–Q plot of effective design delivery mechanisms.

The sampled data distribution of normality shall be tested using before any statistical testing is conducted. Determination of its statistical categories, thus by observing the Shapiro-wilk [28,29] significance level of p value and by observing the frequency distribution histograms or Quantile Quantile(Q-Q) plot we can determine the data distribution. Therefore, based on the value of p = .001, from Table 1 of normality, the sample data is significantly deviated from the normally distributed population, which implies that the data is not normally distributed. On the other hand, by observing the skewness and kurtosis values of the descriptive statistics (Table 2) and the Q-Q plot (Fig. 3) the data is not normally distributed. The data testing technique would be nonparametric.

**Table 1:** Test of normality for effective design delivery mechanisms.

Tests of Normality						
	Kolmogorov-Smirnov^a^			Shapiro-Wilk		
	Statistic	df	Sig.	Statistic	df	Sig.
Effective Design	0.409	204	0.000	0.650	204	0.000

a . Lilliefors Significance Correction.

**Table 2:** Descriptive study of effective design delivery mechanisms.

Descriptive				
			Statistic	Std. Error
Effective Design	Mean		4.5980	0.04424
	95 % Confidence Interval for Mean	Lower Bound	4.5108	
		Upper Bound	4.6853	
	5 % Trimmed Mean		4.6699	
	Median		5.0000	
	Variance		0.399	
	Std. Deviation		0.63183	
	Minimum		2.00	
	Maximum		5.00	
	Range		3.00	
	Interquartile Range		1.00	
	Skewness		−1.440	0.170
	Kurtosis		1.435	0.339

The Spearman rank order correlation is employed on the data considering the data's characteristic of monotonicity (the value of one variable would increase or decrease as the value of the other variables increase or decrease) and the distribution of the data shall also be linearly related. Table 3 shows Spearman's rank order correlation coefficient. Association is measured using the range of r values suggested by Schober et al. [30] as absolute magnitude of the observed correlation coefficient interpretation, 0.00–0.10 Negligible correlation, 0.10–0.39 Weak correlation, 0.40–0.69 Moderate correlation, 0.70–0.89 Strong correlation, 0.90–1.00 Very strong correlation.

**Table 3:** Spearman's rank order correlation coefficient for effective design delivery mechanisms.

Correlations					
			Understanding the design problem, and design constraints	Formulation of effective design objectives, contexts, and concepts	Setting evaluation frameworks in the design process
Spearman's rho	Understanding the design problem, and design constraints	Correlation Coefficient	1.000	0.529^a^	0.302^a^
		Sig. (2-tailed)	.	0.000	0.000
		N	204	204	204
	Formulation of effective design objectives, contexts, and concepts	Correlation Coefficient	0.529^a^	1.000	0.448^a^
		Sig. (2-tailed)	0.000	.	0.000
		N	204	204	204
	Setting evaluation frameworks in the design process	Correlation Coefficient	0.302^a^	0.448^a^	1.000
		Sig. (2-tailed)	0.000	0.000	.
		N	204	204	204

a . Correlation is significant at the 0.001 level (2-tailed).

Table 3 clearly shows that the part of the variables that can be illustrated as an example. Therefore, with 1 % significance level with a range of weak to moderate associations in the same direction. There is a significant moderate correlation between understanding the design problems and design constraints and the formulation of effective design objectives, contexts, and concepts of urban transport infrastructure (r = 0.529, p = 0.001, N = 204). In addition, the correlation between the formulation of effective design objectives, context, and concepts and setting evaluation frameworks in the design process is moderate (r = 0.448, p = 0.001, N = 204). Whereas the variables of understanding the design problem and design constraint and setting evaluation frameworks in the design process have a weak correlation with positive direction (r = 0.302, p = 0.001, N = 204).

#### 3.2.2 Principal component analysis (PCA)

The steps followed for the principal factor analysis are listed below [31].

Step 1 Determine KMO (Kaiser-Meyer-Olkin) and Bartlett's test of sphericity and if the results range within a 5 % significance level and KMO more than 0.500, proceed with principal component analysis.

Step 2 Compute the communality by the given extraction and rotation method and observe the value of extraction shall be more than 0.500.

Step 3 Explain the total cumulative variance of the generated components and they should explain more than 50 % of the total variance of the components and observe the eigenvalue which should be more than 1. It is also good to do parallel analysis by using Monte Carlo PCA simulations to determine which eigenvalues shall be considered.

Step 4 Finally, check the components in the rotated matrix thoroughly if there is any overlapping and crossing over of component factors.

Step 5 Validity and reliability of the component factors shall be checked.

Therefore, identifying the effective attributes for the delivery system has been analyzed using the principal factor analysis (PCA) technique. A data can be considered for PCA analysis if its KMO Measure of Sampling is Adequate (>0.5) and if its Bartlett's Test of Sphericity is significant (<0.05). The test for both requirements was acceptable with a KMO = 0.891 and a significance of sphericity of 0.001 (Table 4).

**Table 4:** KMO and Bartlett's test of sphericity for EDM.

KMO and Bartlett's Test		
Kaiser-Meyer-Olkin Measure of Sampling Adequacy.		0.891
Bartlett's Test of Sphericity	Approx. Chi-Square	4081.630
	df	496
	Sig.	0.000

Analyses of design systems have been analyzed using the varimax with Kaiser Normalization method of rotation. The method of extraction employed was Principal Component Analysis.

The factors identified through the literatures were about 16 and the principal component analysis was used for the exploratory factor study of the effective design factors for urban transport infrastructures. The analysis was conducted using IBM SPSS 20.0 and the rotation technique which influences the correlation of the factors was determined to be orthogonal than the oblique one. The rotation type was Varimax method, which has a significant output on the component loads and extraction using PCA with Kaiser normalization. The factor of ‘Professional certification and level of competency’ was loaded below 0.5 and it was excluded. The other factor, which is ‘Ontime design delivery through performance-based approaches’ loads in to two components and this factor has also been removed since it creates data instability. A total of 14 factors have been extracted as a communality which loads more than a loading of 0.5 (Table 5).

**Table 5:** Communality extraction of design delivery factors.

Communalities		
	Initial	Extraction
Value of understanding effective design delivery system	1.000	0.667
Consideration of input, process, and outcomes of quality design delivery	1.000	0.734
Understanding the design problem, and design constraints	1.000	0.760
Formulation of effective design objectives, contexts, and concepts	1.000	0.662
Setting evaluation frameworks in the design process	1.000	0.475
Understanding urbanity and its complexity	1.000	0.886
Necessity of urban infrastructure standards for design delivery	1.000	0.716
Institutional capacity for evaluation and approval of effective design	1.000	0.667
Professional capacity for effective design delivery	1.000	0.898
Collaboration of professionals in delivering effective designs	1.000	0.654
Coordination and integration between/across institutions	1.000	0.733
Relevance of design competitions, design review and public feed back	1.000	0.523
Accountability of the risk of design output	1.000	0.554
Implementing design tools such as Building Information Modelling (BIM)	1.000	0.671

Extraction Method: Principal Component Analysis.

The extracted factors of communality were investigated in the total variance explained (Table 6), showing that Four components (component 1, component 2, component 3, and Component 4) out of 14 factors have an initial eigenvalue of more than one (1.0) and these components explained more than 68 % of the total variance or common variance of factors.

**Table 6:** Total variance explained for design delivery factors.

Total Variance Explained									
Component	Initial Eigenvalues			Extraction Sums of Squared Loadings			Rotation Sums of Squared Loadings		
	Total	% of Variance	Cumulative %	Total	% of Variance	Cumulative %	Total	% of Variance	Cumulative %
1	5.375	38.396	38.396	5.375	38.396	38.396	2.909	20.776	20.776
2	1.921	13.719	52.115	1.921	13.719	52.115	2.485	17.751	38.527
3	1.207	8.623	60.738	1.207	8.623	60.738	2.312	16.518	55.044
4	1.098	7.841	68.579	1.098	7.841	68.579	1.895	13.535	68.579
5	0.795	5.678	74.258						
6	0.673	4.810	79.068						
7	0.625	4.464	83.532						
8	0.503	3.594	87.125						
9	0.448	3.203	90.329						
10	0.329	2.348	92.677						
11	0.314	2.243	94.920						
12	0.295	2.107	97.026						
13	0.244	1.742	98.768						
14	0.172	1.232	100.00						

Extraction Method: Principal Component Analysis.

**Fig. 4:**
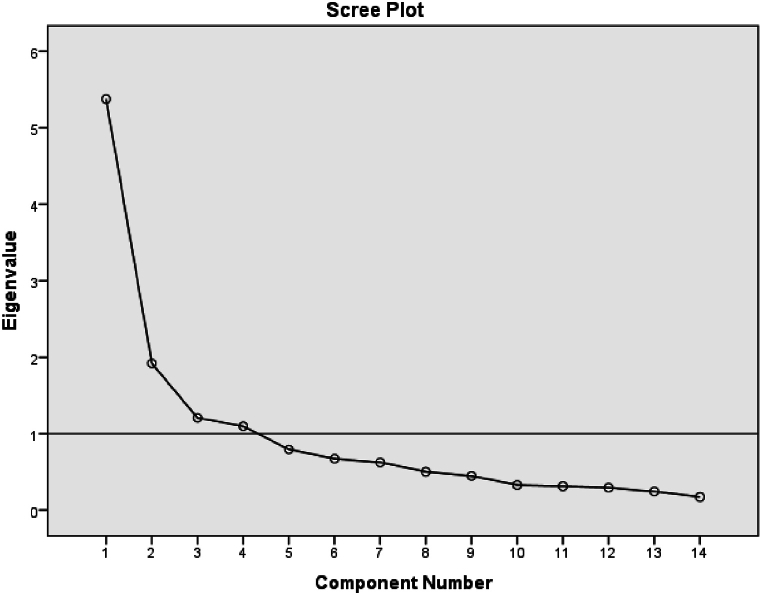
Scree plot for design delivery factors.

The representation of identifying components using the scree plot has also been presented in Fig. 4. The figure justifies the four factors loading eigenvalue of more than one and the rest of the factors are just having a value of less than one.

The result of the scree plot shall be rechecked with parallel analysis of monte Carlo PCA and the simulation signifies the components can be of Four in number since all eigenvalues randomly generated (1.468,1.351,1.270 and 1.195) were lesser values of the three component factors from the scree plot findings of PCA method of extraction (5.375,1.921,1.207 and 1.098). The comparison of the results showed that the eigenvalues of the component number 1 and 2 are greater in the PCA analysis, where the component numbers 3 and 4 have almost the same as that of Monte Carlo PCA for parallel analysis. Therefore, we can take four components. Therefore, the rotated metric of components of effective design delivery parameters are extracted based on the orthogonal rotations of Varimax since this rotation type creates a good orthogonality among the extracted factors (Table 7).

**Table 7:** Rotated component matrix of design delivery factors.

Rotated Component Matrix^a^				
	Component			
	1	2	3	4
Understanding the design problem, and design constraints	0.834			
Consideration of input, process, and outcomes of quality design delivery	0.773			
Formulation of effective design objectives, contexts, and concepts	0.721			
Value of understanding effective design delivery system	0.710			
Coordination and integration between/across institutions		0.804		
Collaboration of professionals in delivering effective designs		0.757		
Relevance of design competitions, design review and public feed back		0.655		
Accountability of the risk of design output		0.589		
Necessity of urban infrastructure standards for design delivery			0.792	
Institutional capacity for evaluation and approval of effective design			0.761	
Implementing design tools such as Building Information Modelling (BIM)			0.636	
Setting evaluation frameworks in the design process			0.500	
Professional capacity for effective design delivery				0.945
Understanding urbanity and its complexity				0.941

Extraction Method: Principal Component Analysis.

Rotation Method: Varimax with Kaiser Normalization.

a . Rotation converged in 6 iterations.

The rotated loadings of factors (Table 7) have been assigned to the four components with a loading of 0.5 and above, which agrees the standard value. Component 1 comprises of four factors of loading from 0.710 to 0.834, and the factors loading on component 2 are four in number and the loadings are from 0.589 to 0.804. Component 3 has also loaded four factors with a loading amount ranging from 0.500 to 0.792, whereas the fourth component loads on two and does not have enough factors, but the loadings are exceptionally good, and they are 0.941 and 0.945.

The latent variables that exist in the effective design delivery systems in urban transport infrastructures are four components and to confirm the component model, a test of reliability and validity of the component factors were conducted. Reliability statistics of the N = 14 items of factors result at Cronbach's alpha of 0.898, which is acceptable since it exceeds the minimum value of 0.7. Discriminant validity is also employed to test the component model by the examination of the component transformation matrix, as shown below (Table 8). Correlations between factors should not exceed 0.7(0.7*0.7 = 0.49 = 49 % of shared variance) [32].

**Table 8:** Component transformation matrix of effective design delivery.

Component Transformation Matrix				
Component	1	2	3	4
1	0.569	0.551	0.495	0.358
2	−0.271	0.774	−0.150	−0.552
3	−0.641	−0.049	0.765	0.037
4	−0.438	0.309	−0.384	0.752

Extraction Method: Principal Component Analysis.

Rotation Method: Varimax with Kaiser Normalization.

**Fig. 5:**
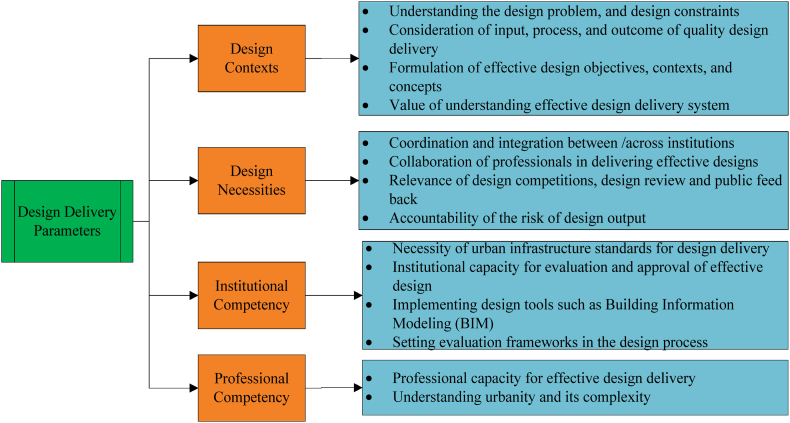
Design delivery parameters for urban transport infrastructures.

Table 8 shows that the correlation matrix of the components which load across the factors is 0.641(41 % shared variance), less than the threshold value of 0.70(49 %) correlation coefficient and the components self-correlation is greater than the cross correlation (0.569 > 0.551 for component 1; 0.774 > 0.552 for component 2; 0.765 > 0.641 for component 3 and 0.752 > 0.438 for component 4). Therefore, the component matrix developed is proved to be a working model of identifying the latent components of the effective design mechanisms in the delivery urban transport infrastructures in Ethiopia. The data including the latent variables can be re-organized as follows in Fig. 5.

The four components of effective design delivery parameters that should be considered in the provision of urban transport infrastructure are design context, necessities, institutions' competency, and professionals’ competency (Fig. 5). All factors should be considered based on their sequence of appearance in the rotated component matrix.

## 4 Discussion

Infrastructure is a long-term asset and is a crucial aspect of industrial and overall economic development, boosting economic growth by increasing productivity, competitiveness, and the living standard of the general population [33,34]. Various sectors theoretically contribute to the economic growth of a country. Agriculture, manufacturing, energy, construction, and other industries are examples. The built environment poses a significant challenge to professionals. Building construction, fit-out, service, and eventual demolition have a significant effect on the environment, both directly (via material and energy use and the resulting emissions and waste) and indirectly (through pressures on inefficient infrastructure). The built environment often has a significant effect on individuals, communities, and organizations' physical and economic health and well-being [35]. A decent infrastructure is a joy to see, and it will benefit a society or organization, as well as professionals’ capacity to learn and maximize their efficiency. A detrimental structure would have the opposite effect. Infrastructure and urban conditions in Ethiopia are unhealthy and unsustainable as they lead to ill health and isolation, disrupt society, and generate undue financial responsibility [36].

The key elements that should be incorporated while the design process is in progress have been highlighted by the researchers through careful investigation. Design contexts, design needs, institutional competency, and professional competency are design parameters to effectively apply a sound design process as a key component. These components have typically been complemented by other factors that aid in critically addressing the design objectives. The contract system that is used, such as design bid build, design build, or integrated project delivery, would be linked to the design practice and its effectiveness [[37], [38], [39]].

### 4.1 Design contexts

Design basically arises from the existential problem that needs a design solution whether in product manufacturing or built environment provision. The principal components found from the reduction of observable variables from factor analysis are four parameters. The first one is about the level of understanding the design problem and the design constraints and value of understanding an effective design delivery system. Context of the design process also considers the input, process, and outcomes of quality design delivery. And finally, the formulation of effective design objectives, contexts, and concepts shall be the main factors to be considered in the provision of effective urban infrastructure.

A clear understanding of the design problems, design constraints, design objective, and concepts towards making delivery efficient would highlight the contextual requirement in the first stage. This articulation of synthesizing core existential and futuristic demands could also be addressed by a standard formulation of effective design schemes. The current design problem in regard to the urban regions of Ethiopia is bound by the rapid population growth and unmanageable urbanization process [36]. Constraints as such economic and political instability need to be considered in the design and delivery of mobility infrastructure. Design contextual factors also needed to be incorporated in the input and output variables categorization of the design delivery system to effectively address and maximize the infrastructure effectiveness.

In the case of complete inputs needed, the necessary methods and processes employed along with the expected design output dictate the quality of effective planning delivery. These phases of planning parameters indicate the contextualization of both the time frame and design objectives. The contexts of environmental situation are both the physical and natural economic system, social system. Therefore, design in context is one of the essential components to address the effective delivery of the design process. Contextual analysis at every stage of infrastructure design shall be the requirement of design assignment.

Bertolini et al. (2005) forwarded that accessibility is determined by the characteristics of the transportation system of walking distance and travel time as well as the characteristics of land use variety (functional mix of activities and densities). And the integration of the two systems indicates how far the desired destination may be reached from a given point of reference. Road network, street design, building density, and land use variety are some of the major characteristics that influence the level of accessibility [15]. Compared to a less developed location, a well-designed metropolitan environment and an efficient transportation system give a better level of accessibility [13]. As illustrated in, the transport system is at the center of accessibility, connecting people to preferred destinations (work, school, entertainment, shopping, and different services) that are functions of a variety of daily uses. Therefore, accessibility is a larger notion that includes mobility, closeness, and connectedness because of individuals interacting with transportation systems and land use activities [13].

### 4.2 Design necessities

The current design process for Ethiopian transportation infrastructure does not consider the necessary elements in the design assignment [36,40]. Consideration of these design necessities would help to enhance the designer's efficiency to increase the performance of the infrastructure [41].

In regions of growing economies like Ethiopia, the provision of efficient transportation infrastructure is a fundamental function of design as a project delivery instrument. The population increase and rapid urbanization of city regions are two large challenges in the design process. The design demands, which have an impact on this delivery procedure, are one of the key factors that must be carefully considered.

The design necessity component comprises of four observed factors addressing the design requirements in urban infrastructure provision. Coordination and integration between institutions and collaboration of professionals in delivering effective design and the basic requirements of effective design delivery. In addition, the relevance of design competitions, design review, and public feedback are important criteria for effective design delivery. Accountability of the risk of design output is also a determining factor.

When designing transportation infrastructure, it is crucial to recognize the value of effective delivery methods. The degree of general understanding of effective delivery and how well a design task is carried out will greatly impact the process' effectiveness. However, having the knowledge, expertise, and professional capacity to make effective design work while considering design requirements outweighs it. Prior consideration should be given to professional collaboration to achieve effective design.

The coordination and integration between and across stakeholders or sectors are a necessity to be considered during the design delivery. In addition, practical collaboration of professionals in delivering effective designs is one of the key factors required to be fulfilled in the design work. Professionals of diverse expertise would help to address the design objective of efficient infrastructure provision. The study suggested that the involvement of architects, landscape architects, urban designers, highway engineers, structural engineers, bridge engineers, material engineers, and environmentalists should participate in the realization of the project. By then, accountability of the risk of design output could be considered for minimizing risk of infrastructure design.

Infrastructure helps mitigate the drawbacks of density, but it is uncertain whether infrastructure becomes appealing as institutional quality improves. The essential condition is whether limitations on harmful conduct improve the stock of effective infrastructure, hence reducing the need to spend more, or whether new infrastructure increases the marginal gains [4].

Relevance of design competitions, design review, and public feedback of urban infrastructure design are the criteria most institutions residing in the federal or national level failed to practice due to lack of awareness of its relevance. Design is a result of creativity in its essence and this process shall be conducted through an in-house design competition launched with a wider audience of experts and designers in the industry. The design review work is also a valuable step to achieve effective design output. The review process shall also include public feedback since the end user is the sole owner of the designed infrastructure. The overall design competition and design review process shall also be standardized through code of practice and frameworks since it is one of the critical design necessities.

### 4.3 Institutional competencies

Institutions are defined as those that are authorized to design and supervise with certified competencies in the design process of transportation infrastructure and are required by law to manage roads and railway infrastructure. The institutions are the Ethiopian Roads Administration, Addis Ababa City Roads Authority, and consulting firms.

Under the institutional competency major factor, the study outlines four factors. To properly incorporate the desired relevant aspects, the institutional capability for evaluation and approval of an effective design would be the determining factor. Urban infrastructure standards are essential for delivering designs and establishing frameworks for evaluation during the design process. The use of design tools like Building Information Modelling (BIM) must be taken into account when developing urban transportation infrastructure. It is quite intriguing how the introduction of guiding standards, design tools, and evaluation frameworks into the infrastructure design process was one of the primary causes that led to a preceding suggestion.

Institutional competencies which focus on the situational systems of institutions which devise strategies and action plans of the institutional duties of the urban transport infrastructure. Institutions traditionally launch, monitor lead, and approve the design assignments to the respective stakeholder. The study peculiarly suggested that the institutions should increase their design efficiency and capacity to devise mechanisms, guidelines, and standards at all stages of the design process of urban infrastructure. Therefore, the institutional capacity for evaluation and approval of effective design at every stage of the process would enhance the provision of urban infrastructure.

The defined design delivery necessitates the sound urban infrastructure standard. These standards would help to facilitate the transport infrastructure through guidance, assessment, and evaluation systems. The main evaluation system shall also be done using frameworks. Whereas the implementation process can also be supported by the consideration of design tools such as Building Information Modelling (BIM). BIM by its very practicality to holistically monitor, evaluate and approve the design product in its life cycle. The information management of design assignment is stored in a database whoever the institution member can get enough of the design solution. For this such system of information management, the institution shall be ready to capacitate its staff and infrastructure facilities.

Institutions readiness to incorporate institutional competencies to evaluate and approve design products and the necessity of standards, design tools, and frameworks would facilitate the effective design delivery. The factors aligned with this component of the effective parameters would highly fill the gap in the consideration of institutional issues towards project delivery competency in the design process. However, this component alone would not be enough to achieve sustainable and effective practice. Along with institutional capacity, consideration of professionals’ competencies has also played a great role in making effective design delivery.

Most of the physical infrastructure is fixed and delivered via networks and nodes [10]. Interoperability within each mode, but not necessarily between modes, is one of the distinguishing characteristics of physical infrastructure. Physical infrastructure serves primarily economic purposes but also contributes to social and environmental well-being [10]. As stated by Morphet (2016), integration is the primary method for effective infrastructure delivery. Before exploring how networks, nodes, and nodes may be brought together at different geographical scales, it is critical to understand how this integration works in practice through implementation time frames and delivery methods. The integration of transportation infrastructure and land use is central to the planning system and its role in delivering efficient, effective, and sustainable locations.

According to Christopher, Eric, & Amer [42], the process of creating more sustainable transportation also require the creation of four pillars: *'effective governance of land use and transportation; fair, efficient, stable* funding*; strategic infrastructure investments; and attention to neighborhood design’.* These pillars of consideration would result in more effective transportation infrastructure provision. Christopher et al. (2005) attempted to address the critical aspects of governance, finance, strategy, and spatial design for the effective delivery of sustainable transportation. Some of the performance measures of transportation systems are accessibility, health and safety, cost effectiveness, impact on competitiveness, and generation of wealth, consumption of natural capital and production of pollutants [42].

### 4.4 Professional competencies

Competency of the professionals engaged in the transport infrastructure is one of the principal parameters to deliver effective provision of urban infrastructure at the design phase. Unless this factor is properly considered in the design of infrastructure, it would end up being an inefficient transport infrastructure. First, the capacity to understand the value of the sector in rendering urban connectivity in and out of the cities in the margin of the economy is essential. Professionals shall be equipped with the recent advancement of design delivery techniques. At individual and at group level, the awareness of design inputs, processes, methods, and output design delivery process of urban transport infrastructure. Therefore, the professional capacity of designers plays a key factor to determine the level of effectiveness of the infrastructure design.

The other factor that is aligned with the professional competencies is the value of understanding the concept of an urban region and its dynamicity and the extent of its complexity. Designing urban infrastructure in the context of rapid urbanization and population growth would greatly need well-informed competent professionals, having notable qualifications and experience in the area. The case of understanding the concept of urbanization should not be set aside to architects and urban designers or planners, rather all stakeholders involved in the design of such infrastructure shall be acquainted with the concept.

One way of attaining the capacity of professionals in the delivery of sound urban infrastructure in Ethiopian cities would be through proper training and professional career development. According to Blachnicka [43], in the current, unstable environment, adopting a proactive attitude focused on professional development is necessary, and both objective and subjective indicators are used to assess the advancement of a professional career.

Effective urban infrastructure provision is one of the components of sustainable urban transportation. This efficiency in sustainable mobility can be achieved by considering transportation governance, transportation finance, neighborhood spatial design, and infrastructure investment at the same time. The efficacy of transportation infrastructure cannot be addressed by creating new roads and railways; instead, it is important to explore making better use of existing infrastructure rather than developing new [44,45].

Integration, on the other hand, influences the successful provision of transportation infrastructure. Sectoral coordination in the development of urban infrastructure and service supply is crucial in defining the economic and physical growth of cities, as well as the livelihood of city inhabitants, the efficiency and effectiveness of service providers, and the development of any country [46]. Furthermore, [46], stated that the current governance arrangements in terms of institutional arrangements, financial arrangements, and regulatory frameworks were found to be less effective and unsuitable for a multifactor system, in this case, coordination among interdependent infrastructure sectors. As a result, integration within the sector among responsible actors of transport infrastructure, as well as cooperation across sectors such as electricity, water and sewerage, and telecommunications, are required for the successful delivery of roads and railways in Ethiopia. The ever-increasing demand in this sector must be met with an efficient planning and implementation approach.

## 5 Conclusion

The need for efficient transport infrastructure in urban areas is inevitable in a time of large population mobilization from rural to urban regions. The urbanization process and the growth of population in the cities demand a guiding instrument for the proper delivery of social services through a convenient transportation system. These systems require efficient infrastructure of roads, railways, and bridges. Effective design delivery tools are supported in the study through a literature review. It is also noted that effective design delivery approaches of transport infrastructures are meant to be effective parameters to measure how transport infrastructures such as roads and railways function to deliver the intended purpose.

Several variables were identified to be effective parameters through the literature review, and these large parameters were structured to be rated by the respondents using a structured survey questionnaire. The respondents in the design of transport infrastructures in general have been randomly selected to fill out the questionnaire. The responses gathered were analyzed using a factorial analysis method of data reduction was employed. The technique to reduce the factors was principal component analysis.

The factors that load at a significant level were grouped into components. These four components were latent in characteristics but had an observed factor that directly impacts the design and delivery process of urban transport infrastructure. The principal components extracted are design context, design necessities, institutional competency, and professional competency. The consideration of these main parameters at the time of design assignment would facilitate the design products' effectiveness. Urban transport infrastructure, in the case of Ethiopia, has multitasked activities from the design conception to the final design of construction methodology preparation. The context, necessity, and professional and institutional competencies must all be taken into consideration during the design process.

The study is focused on the exploratory phase of the research, and a confirmatory factor analysis might be used to develop further measurement models. To comprehend the covariance extents of the hidden components explained, it would be helpful to consider the context of data reduction of large variables and the pattern or path analysis. The design methods used for transportation infrastructure including roads, bridges, and railroads are equally relevant to the subject. If the transportation services industry were included in the survey study, the case might be more inclusive.

## Funding

There is no potential funding source.

## Data availability

Data will be made available on request.

## CRediT authorship contribution statement

**Leule M. Hailemariam:** Writing – original draft, Investigation, Formal analysis, Conceptualization. **Denamo A. Nuramo:** Writing – review & editing, Supervision, Conceptualization.

## Declaration of competing interest

The authors declare that they have no known competing financial interests or personal relationships that could have appeared to influence the work reported in this paper.
