# Supplementary material for: Factor analysis of key parameters for effective design delivery of urban transport infrastructure in Ethiopia
Source: Heliyon. 2024 Jul 15;10(14):e34681. doi: 10.1016/j.heliyon.2024.e34681 (PMC11315186; doi:10.1016/j.heliyon.2024.e34681)
Supplement: Multimedia component 1 [file mmc1.pdf]

SUSTAINABLE URBAN BRIDGE DESIGN IN ETHIOPIA (A SURVEY)

This is a Ph.D. study to develop a multi-criteria framework for designing a sustainable urban bridge at Addis Ababa University. You are invited to participate in the study by completing the following survey which will take approximately 25 minutes to complete. The questionnaire has five sections: **Section A**: Background information, **Section B**: Identification of effective planning and design delivery mechanisms of urban transport infrastructures, **Section C**: Application of sustainability principles in the urban bridge design process, **Section D**: Development of sustainable urban bridge design criteria for multi-criteria framework and **Section E**: Identification of key roles of a multi-criteria framework for sustainable urban bridge design.

SECTION A: BACKGROUND INFORMATION

Please tick the appropriate box or fill in the blank space when answering the questions.

1) **Gender:** Male ☐ Female ☐
2) **Higher Level of Education:** Ph.D. ☐ Master's ☐ Bachelor's ☐
3) **Specialization based on education and/or experience** *[you can tick in one box]*:

▪ Architecture/Urban design	☐		▪ Railway engineering	☐
▪ Bridge engineering	☐		▪ Highway engineering	☐
▪ Structural engineering	☐		▪ Planning	☐

Other (please specify): ______________________________

4) **Design Experience** *[you can tick in one box]*:

▪ Buildings	☐		▪ Railway	☐
▪ Bridges	☐		▪ Highway	☐
▪ Landscape	☐		▪ Infrastructure (any other)	☐

Other (please specify): ______________________________

5) **Years of experience in design in general:**

Less than 6	☐		6-10	☐		11-20	☐		More than 20	☐

6) **Years of experience in bridge infrastructure design in particular:**

Less than 6	☐		6-10	☐		11-20	☐		More than 20	☐

7) **In how many design projects have you participated as a designer in your professional practice?**

Less than 6	☐		6-10	☐		11-20	☐		More than 20	☐

SECTION B. IDENTIFICATION OF EFFECTIVE PLANNING AND DESIGN DELIVERY MECHANISMS OF URBAN TRANSPORT INFRASTRUCTURES (Please tick/click from the options where applicable)

Note: *Effective Delivery Mechanisms (EDM) of transport infrastructures are meant to be effective parameters to measure how the transport infrastructures such as roads and railways function to deliver the intended purpose.*

Rate on a scale of 1 to 5 the following parameters in terms of their importance in the identification of effective mechanisms about delivering urban transport infrastructure under which they are listed (1= not important, 2= low importance, 3=neutral, 4= important & 5 = very important).

	**Parameters**	**1**	**2**	**3**	**4**	**5**
	**1. Planning Delivery Mechanisms**					
a	Value of understanding effective delivery system through planning	☐	☐	☐	☐	☐

b	Consideration of input, process, and outcome of quality planning delivery	☐	☐	☐	☐	☐
c	Contextual considerations	☐	☐	☐	☐	☐
d	Existential considerations	☐	☐	☐	☐	☐
e	Futuristic and forecasting considerations	☐	☐	☐	☐	☐
f	Integration between stakeholders	☐	☐	☐	☐	☐
g	Collaboration among professionals	☐	☐	☐	☐	☐
h	Coordination across sectors	☐	☐	☐	☐	☐
i	A holistic view of planning delivery mechanisms	☐	☐	☐	☐	☐
j	Sustainability considerations	☐	☐	☐	☐	☐
k	Institutional governance issue	☐	☐	☐	☐	☐
l	Professional capacity and competency	☐	☐	☐	☐	☐
m	Institutional capacity	☐	☐	☐	☐	☐
n	Financial sources availability	☐	☐	☐	☐	☐
o	Private participation in the delivery of transport infrastructures	☐	☐	☐	☐	☐
p	The necessity of standards for planning delivery	☐	☐	☐	☐	☐
	**2. Design Delivery Mechanisms**					
a	Value of understanding effective design delivery system	☐	☐	☐	☐	☐
b	Consideration of input, process, and outcome of quality design delivery	☐	☐	☐	☐	☐
c	Understanding the design problem, and design constraints	☐	☐	☐	☐	☐
d	Formulation of effective design objectives, contexts, and concepts	☐	☐	☐	☐	☐
e	Setting evaluation frameworks in the design process	☐	☐	☐	☐	☐
f	Understanding urbanity and its complexity	☐	☐	☐	☐	☐
g	The necessity of urban infrastructure standards for design delivery	☐	☐	☐	☐	☐
h	Institutional capacity for evaluation and approval of the effective design	☐	☐	☐	☐	☐
i	Professional capacity for effective design delivery	☐	☐	☐	☐	☐
j	Professional certification and level of competency	☐	☐	☐	☐	☐
k	Collaboration of professionals in delivering effective designs	☐	☐	☐	☐	☐
l	Coordination and integration between /across institutions	☐	☐	☐	☐	☐
m	Relevance of design competitions, design review, and public feedback	☐	☐	☐	☐	☐
n	Online design delivery through a performance-based approach	☐	☐	☐	☐	☐
o	Accountability of the risk of design output	☐	☐	☐	☐	☐
p	Implementing design tools such as Building Information Modelling (BIM)	☐	☐	☐	☐	☐

SECTION C. APPLICATION OF SUSTAINABILITY PRINCIPLES IN URBAN BRIDGE DESIGN PROCESS (Please tick/click from the options where applicable)

Note: *Sustainable design is an alternative approach to traditional/conventional design which leads toward a less consumptive mindset that embraces global interdependence, environmental stewardship, social responsibility, economic viability, and technologically systematic and considers the impacts of design choices at local, regional, and global levels.*

1. On a scale of 1-5, rate the following statements that best represent your opinion/perception of sustainability based on your bridge design practice (1= strongly disagree,2=disagree, 3= neutral, 4= agree & 5 = strongly agree).

**Statements of perception**		**1**	**2**	**3**	**4**	**5**
a	The bridge design process should include sustainability considerations	☐	☐	☐	☐	☐
b	Guidelines or frameworks for sustainable design shall be easily found in Ethiopia.	☐	☐	☐	☐	☐
c	Sustainability considerations are mainly for satisfying mandatory requirements.	☐	☐	☐	☐	☐
d	Adopting sustainable design practices should be voluntary.	☐	☐	☐	☐	☐

e	The use of sustainable design principles, processes, and methods will help to preserve natural resources.	☐	☐	☐	☐	☐
f	I am aware that sustainability is getting more recognition among my colleagues and co-workers.	☐	☐	☐	☐	☐
g	I believe that using sustainable design principles will increase construction costs and time.	☐	☐	☐	☐	☐
h	The use of sustainable design principles would reduce construction costs and time.	☐	☐	☐	☐	☐
i	Even if there is an increase in construction cost and time, I have noticed that my colleagues intended to incorporate sustainability in bridge design practice.	☐	☐	☐	☐	☐
j	Even if there is an increase in construction cost and time, I have noticed that my clients intended to apply sustainable design principles, processes, and methods in projects.	☐	☐	☐	☐	☐
k	Important for Planners, Architects, and Engineers to be conscious that some of the designs they execute have an impact on the environment.	☐	☐	☐	☐	☐
l	Important for Planners, Architects, and Engineers to be conscious that some of the designs they execute have an impact on the economy.	☐	☐	☐	☐	☐
m	Important for Planners, Architects, and Engineers to be conscious that some of the designs they execute have an impact on society.	☐	☐	☐	☐	☐
n	Important for Planners, Architects, and Engineers to be conscious that some of the designs they execute have an impact on the institution's performance.	☐	☐	☐	☐	☐
o	Important for Planners, Architects, and Engineers to be conscious that some of the designs they execute have an impact on technological usage.	☐	☐	☐	☐	☐

2. Based on your experience, please rate the following (on a scale of 1-5) how they affect your sustainable design practices (very low=1, low=2, moderate =3, high=4 & very high =5).

**Statements**		**1**	**2**	**3**	**4**	**5**
a	Lack of awareness of sustainability	☐	☐	☐	☐	☐
b	Weakness of professionalism	☐	☐	☐	☐	☐
c	Limited finance for sustainable design practice	☐	☐	☐	☐	☐
d	Demands lots of skill and time in conceptualizing, analysing & designing	☐	☐	☐	☐	☐
e	Problems in determining main attributes	☐	☐	☐	☐	☐
f	Lack of access to current and relevant information	☐	☐	☐	☐	☐
g	Inadequate current design facilities and techniques	☐	☐	☐	☐	☐
h	Inadequate instructions about design input, process, and outputs	☐	☐	☐	☐	☐
i	Lack of sustainable design codes and standards	☐	☐	☐	☐	☐
j	Problems in understanding sustainable design processes and criteria	☐	☐	☐	☐	☐
k	Lack of structured protocols or frameworks	☐	☐	☐	☐	☐
l	The conventionality of the current design practice	☐	☐	☐	☐	☐
m	Demanding high effort	☐	☐	☐	☐	☐

3. For each of the following stakeholders, indicate how much influence each has in sustainable urban bridge design on a scale of 1-5, (very low=1, low=2, moderate =3, high=4 & very high =5)

**Stakeholders**		**1**	**2**	**3**	**4**	**5**
a	The client (private and government)	☐	☐	☐	☐	☐
b	Architect /Urban designer	☐	☐	☐	☐	☐
c	Urban Planner	☐	☐	☐	☐	☐
d	Environmental planner	☐	☐	☐	☐	☐
e	Bridge engineer	☐	☐	☐	☐	☐
f	Structural engineer	☐	☐	☐	☐	☐
g	The public	☐	☐	☐	☐	☐

4. What obstacles currently prevent you from practicing a sustainable design of urban bridges in your design assignment? Please rate on a scale of 1-5 (very low=1, low=2, moderate =3, high=4 & very high =5).

**Obstacles**		**1**	**2**	**3**	**4**	**5**
a	Lack of information on sustainable design concepts	☐	☐	☐	☐	☐
b	The problem in evaluating information	☐	☐	☐	☐	☐
c	Uncertainty in the liability for final works	☐	☐	☐	☐	☐
d	Lack of codes and standards	☐	☐	☐	☐	☐
e	Lack of tools and data to evaluate sustainable design practice	☐	☐	☐	☐	☐
f	Perception of the extra cost being incurred	☐	☐	☐	☐	☐
g	Perception of extra time being incurred	☐	☐	☐	☐	☐
h	Difficulties in balancing environmental, institutional, technological, economic & social issues	☐	☐	☐	☐	☐
i	The perception that sustainable design is related to environmental design	☐	☐	☐	☐	☐
j	Aesthetically less pleasing	☐	☐	☐	☐	☐
k	Possible delay delivery due to sustainability requirement	☐	☐	☐	☐	☐
l	Limited availability and reliability of design tools	☐	☐	☐	☐	☐
m	Low flexibility for alternatives or substitutes of the design process	☐	☐	☐	☐	☐
n	Unwillingness to change the conventional way of designing	☐	☐	☐	☐	☐
o	Lack of support from officials’ and or Politicians	☐	☐	☐	☐	☐
p	Lack of professionals who are equipped with the concept	☐	☐	☐	☐	☐

5. What benefits are available for practicing a sustainable design of urban bridges in your design assignment? Please rate on a scale of 1-5 (very low=1, low=2, moderate =3, high=4 & very high =5).

**Benefits**		**1**	**2**	**3**	**4**	**5**
a	Helps to holistically approach a design problem	☐	☐	☐	☐	☐
b	Addressing the major resource consumption problem efficiently	☐	☐	☐	☐	☐
c	Reduce the environmental impact of construction works of a design product	☐	☐	☐	☐	☐
d	Increase social benefits	☐	☐	☐	☐	☐
e	Utilize the recent technologies and the state of the art of design approach	☐	☐	☐	☐	☐
f	Optimize economy	☐	☐	☐	☐	☐
g	Introduce a good institutional culture for delivering efficient projects	☐	☐	☐	☐	☐
h	Mitigate climatic problems	☐	☐	☐	☐	☐
i	Possibility to increase professional collaboration	☐	☐	☐	☐	☐
j	Facilitate sound issuance, approval, and monitoring institutional capacity	☐	☐	☐	☐	☐

SECTION D. DEVELOPMENT OF SUSTAINABLE URBAN BRIDGE DESIGN CRITERIA FOR MULTI-CRITERIA FRAMEWORK (Please tick/click the options where applicable)

Note: *A multi-criteria framework is a framework that includes numerous criteria of a relationship between broad use of difficult-to-understand ideas, concepts, and methodologies.*

Rate on a scale of 1 to 5 the following criteria in terms of their importance in the sustainable urban bridge design and about the sustainability categories under which they are listed. (1= not important, 2=low importance,3= neutral, 4= important & 5 = very important).

**Criteria**		**1**	**2**	**3**	**4**	**5**
**1. Social criteria**						
a	Public access	☐	☐	☐	☐	☐
b	Cultural heritage	☐	☐	☐	☐	☐
c	Public perceptions	☐	☐	☐	☐	☐
d	Vehicle operation cost	☐	☐	☐	☐	☐
e	Driver delay cost	☐	☐	☐	☐	☐
f	Safety cost	☐	☐	☐	☐	☐

g	Driver comfort	☐	☐	☐	☐	☐
**2. Environmental criteria**						
a	Potential for recycling and reuse of design materials	☐	☐	☐	☐	☐
b	Availability of environmentally sound disposal options and waste management techniques	☐	☐	☐	☐	☐
c	Impact of material on air quality	☐	☐	☐	☐	☐
d	Ozone depletion potential	☐	☐	☐	☐	☐
e	Environmental impact during material production, transportation, and construction	☐	☐	☐	☐	☐
f	Zero or low toxicity and acidity of materials	☐	☐	☐	☐	☐
g	Environmental statutory compliance	☐	☐	☐	☐	☐
h	Minimize pollution (air, land, water, etc)	☐	☐	☐	☐	☐
i	Traffic load, speed limit, noise,	☐	☐	☐	☐	☐
j	Land use, site area and access to the site, topography	☐	☐	☐	☐	☐
k	Environmental adaptability	☐	☐	☐	☐	☐
l	Create visual impact	☐	☐	☐	☐	☐
m	Global warming impact	☐	☐	☐	☐	☐
**3. Economic criteria**						
a	Use of local material	☐	☐	☐	☐	☐
b	Initial cost	☐	☐	☐	☐	☐
c	Production cost	☐	☐	☐	☐	☐
d	Construction cost (direct and indirect cost)	☐	☐	☐	☐	☐
e	Maintenance cost	☐	☐	☐	☐	☐
f	Life cycle cost (initial cost, maintenance cost, repair cost, etc)	☐	☐	☐	☐	☐
g	Energy-saving and thermal insulation	☐	☐	☐	☐	☐
**4. Institutional criteria**						
a	Design Procurement method	☐	☐	☐	☐	☐
b	Quality assurance mechanisms	☐	☐	☐	☐	☐
c	Level of workers skill	☐	☐	☐	☐	☐
d	Standardized Project administration (planning, design, and construction)	☐	☐	☐	☐	☐
e	Ethical considerations during design delivery of the project	☐	☐	☐	☐	☐
f	Considerations of occupational health and safety	☐	☐	☐	☐	☐
g	Public health and safety measures	☐	☐	☐	☐	☐
h	Suitability of project delivery systems	☐	☐	☐	☐	☐
i	The capacity of approval, follow up and monitoring institutions and personnel	☐	☐	☐	☐	☐
**5. Technical and Technological criteria**						
**5.1 Aesthetic/Form**						
a	Clear structural system, symmetry, simplicity	☐	☐	☐	☐	☐
b	Good proportions, balance, character, rhythm, continuity, scale, lighting	☐	☐	☐	☐	☐
c	Good order, mass, sincerity, uniqueness, Coloring, depth	☐	☐	☐	☐	☐
d	Integration into environment	☐	☐	☐	☐	☐
e	Choice of materials	☐	☐	☐	☐	☐
f	Space under and above the bridge	☐	☐	☐	☐	☐
g	The recognizable flow of forces	☐	☐	☐	☐	☐
h	Conducting design competition	☐	☐	☐	☐	☐
**5.2 Functional**						
a	Proper feasibility study	☐	☐	☐	☐	☐
b	Conducting design competition	☐	☐	☐	☐	☐
c	Bridge geometry, span, geometric design	☐	☐	☐	☐	☐

d	Durability, strength, safety, thermal influence	☐	☐	☐	☐	☐
e	Load and impact resistance, distortion resistance, deformation adaptability	☐	☐	☐	☐	☐
f	Ease of construction, constructability, inspection, and maintenance,	☐	☐	☐	☐	☐
g	Design complexity, construction complexity, erection complexity	☐	☐	☐	☐	☐
h	Production time, speed of construction, and construction period	☐	☐	☐	☐	☐
i	Materials availability, resource type, reusability, recyclability, replaceability	☐	☐	☐	☐	☐
**5.3 Technological**						
a	Advanced, modern, and appropriate technology	☐	☐	☐	☐	☐
b	Level of dependence on imported technologies	☐	☐	☐	☐	☐
c	Design technologies	☐	☐	☐	☐	☐
d	Construction technologies	☐	☐	☐	☐	☐
e	Erection technologies	☐	☐	☐	☐	☐
f	Deconstruction technologies	☐	☐	☐	☐	☐
g	Assembly technologies	☐	☐	☐	☐	☐
h	Maintenance technologies	☐	☐	☐	☐	☐
i	Usage of Building Information Modelling (BIM) tools	☐	☐	☐	☐	☐

SECTION E. IDENTIFICATION OF KEY ROLE OF MULTI-CRITERIA FRAMEWORK FOR SUSTAINABLE URBAN BRIDGE DESIGN (Please tick/click the options where applicable)

Rate on a scale of 1 to 5 the following key roles degree of importance in the sustainable urban bridge design in Ethiopia (1= not important, 2=low importance,3= neutral, 4= important & 5 = very important).

**Key roles**		**1**	**2**	**3**	**4**	**5**
a	Ability to comprehend design principle, process, and method	☐	☐	☐	☐	☐
b	Creating easiness to the decision process of utilizing resources	☐	☐	☐	☐	☐
c	Possible skill is demanding to utilize the framework	☐	☐	☐	☐	☐
d	Makes the design process complicated	☐	☐	☐	☐	☐
e	Outlines the inputs and goals of design work.	☐	☐	☐	☐	☐
f	Understand and incorporate sustainability principles	☐	☐	☐	☐	☐
g	Guides towards a holistic view of the design objective	☐	☐	☐	☐	☐
h	Helps to assess and evaluate design outputs	☐	☐	☐	☐	☐
i	Incur additional inputs to the design work	☐	☐	☐	☐	☐
j	Mitigate the potential recurring problems of the economy	☐	☐	☐	☐	☐
k	Mitigate the potential recurring problems of social	☐	☐	☐	☐	☐
l	Mitigate the potential recurring problems of institutional	☐	☐	☐	☐	☐
m	Mitigate the potential recurring problems of environmental	☐	☐	☐	☐	☐
n	Mitigate the potential recurring problems of technical and technological	☐	☐	☐	☐	☐

This is the end of the survey. Thank you very much for your time.
